# Rigor and reproducibility instruction in academic medical libraries

**DOI:** 10.5195/jmla.2022.1443

**Published:** 2022-07-01

**Authors:** Fred Willie Zametkin LaPolla, Caitlin J. Bakker, Nina Exner, Tisha Montnech, Alisa Surkis, Hao Ye

**Affiliations:** 1 fred.lapolla@nyulangone.org, Research and Data Librarian, Lead of Data Education, NYU Health Sciences Library, NYU Langone Health, New York, NY; 2 cjbakker@umn.edu, Director, Open Research & Publishing, University of Minnesota Libraries, Minneapolis, MN; 3 nexner@vcu.edu, Research Data Librarian, VCU Libraries, Virginia Commonwealth University, Richmond, VA; 4 tdmentne@ncsu.edu, Research Librarian for Life Sciences and Research Metrics, North Carolina State University Libraries, Raleigh, NC; 5 alisa.surkis@nyulangone.org, Deputy Director, Medical Library, NYU Health Sciences Library, NYU Langone Health, New York, NY; 6 haoye@ufl.edu, Reproducibility Librarian, George A. Smathers Libraries, University of Florida, Gainesville, FL

**Keywords:** reproducibility, replicability, library instruction, education, rigor and reproducibility, NIH requirements

## Abstract

**Background:**

Concerns over scientific reproducibility have grown in recent years, leading the National Institutes of Health (NIH) to require researchers to address these issues in research grant applications. Starting in 2020, training grants were required to provide a plan for educating trainees in rigor and reproducibility. Academic medical centers have responded with different solutions to fill this educational need. As experienced instructors with expertise in topics relating to reproducibility, librarians can play a prominent role in providing trainings, classes, and events to educate investigators and trainees, and bolstering reproducibility in their communities.

**Case Presentations:**

This special report summarizes efforts at five institutions to provide education in reproducibility to biomedical and life sciences researchers. Our goal is to expand awareness of the range of approaches in providing reproducibility services in libraries.

**Conclusions:**

Reproducibility education by medical librarians can take many forms. These specific programs in reproducibility education build upon libraries' existing collaborations, with funder mandates providing a major impetus. Collaborator needs shaped the exact type of educational or other reproducibility support and combined with each library's strengths to yield a diversity of offerings based on capacity and interest. As demand for and complexity of reproducibility education increases due to new institutional and funder mandates, reproducibility education will merit special attention.

## BACKGROUND

Over the past ten years, researchers have become increasingly aware of challenges relating to reproducibility in science [1]. The irreproducibility of research results has also garnered considerable attention in the general press [2–4]. Scholarly journals have published a number of reports and editorials on definitions, issues, and challenges in reproducibility [5–8]. In a 2016 survey from *Nature*, 80% of survey respondents identified better teaching and 90% indicated improved mentorship as ways to address issues in reproducibility [1]. These results suggest that educational programs that directly target research practices could be beneficial in improving reproducibility.

Research agencies have also taken action to promote and increase reproducibility. Most significantly for health sciences libraries, in late 2015, the National Institutes of Health (NIH) announced they would require research grant applications to address rigor and reproducibility starting in 2016 [9]. At this time, NIH also announced that requirements would be forthcoming for instruction on rigor and reproducibility to training grant applicants, though those requirements were not announced until December of 2019 [10]. In 2018, the National Academies of Science released a white paper detailing challenges to quantifying and identifying solutions relating to experimental replicability and computational reproducibility [11].

While definitions abound as to the meaning and scope of reproducibility [5–8, 12], that is not the focus of this paper. In this paper, we use “reproducibility” in a broad sense to include what the National Academy of Science groups under both reproducibility and replicability. We define reproducibility as including

- Designing research projects to be repeatable with consistent results, also called experimental replicability
- Conducting analyses of data and computer simulations so that they may be rerun with consistent results, also called computational reproducibility
- Any additional criteria spelled out by the NIH in its Rigor and Reproducibility framework, for example, addressing sex as a biological variable [13].

Reproducibility instruction has gained increasing attention in the biomedical sphere due to NIH requirements and attention in the general press [14]. The literature includes a discussion of teaching rigor and reproducibility to first-year medical students [15], a report on teaching to neuroimaging students [16], and a gamified approach to teaching reproducibility to medical students [17]. The literature reflects teaching reproducibility in statistics and data science programs [14], as well as in social science classes at the undergraduate level [18–22].

Librarians are often well-positioned to support reproducibility because of existing, established librarian roles in data management, technology instruction, literature searching and appraisal, and open science. Previous papers have described other aspects of library support for reproducibility [23–26], but this paper will focus on support for reproducibility in an educational context in the health sciences. In other words, pedagogy involving reproducibility as a subject matter, not the reproducibility of educational interventions. In describing work on-going at five institutions, our goal is not to prescribe what work should be done at libraries interested in providing reproducibility services, but rather to highlight a variety of approaches and underscore that there is no “one size fits all” approach to teaching about reproducibility. Our hope is that others who are interested in supporting reproducibility may adopt or adapt these models.

## CASE PRESENTATIONS

For each case, we will describe the institutional environment, how the library came to be involved in rigor and reproducibility education, the nature of each library's educational offerings, any available assessment data, and a summary of lessons learned. Contextual information about each library's parent institution is provided in Table 1.

**Table 1:** Demographics of participating institutions Institution Location Carnegie

Institution	Location	Carnegie Class	Staff/Student FTE
NYU Langone Health, Grossman School of Medicine	New York City	Private, Not-for-Profit	167 professors, 1,618 residents/fellow s, 287 PhD candidates, 90 MD/PhD candidates, 539 MD candidates [27]
University of Utah	Salt Lake City, UT	Public	Over 20,000 faculty/staff; 498 medical students
University of Florida	Gainesville, FL	Public	26,500 faculty and staff and more than 7,500 students and residents in the health sciences
University of Minnesota	Minneapoli s and Duluth, MN	Public	Nearly 3,000 faculty, over 7,000 students in the health sciences
Virginia Commonwealt h University	Richmond, VA	Public	1,175 faculty, 759 MD candidates, 281 master's degree, 123 PhD students

## NYU LANGONE HEALTH SCIENCES LIBRARY

### Environment

New York University (NYU) Langone Health Sciences Library primarily serves the NYU Langone Health community, which includes undergraduate and graduate medical education, graduate biomedical sciences education, researchers, and clinicians (See Table 1 for figures).

### Development

In 2015, our academic health sciences library was at an early phase in developing data services, and our educational programming was limited to training in research data management (RDM). Following a sparsely attended RDM training for basic science graduate students, the administration for our doctoral program expressed concern that there was an unfilled need for training in certain essential research skills. After discussions with the program director and leading a focus group of graduate students, we developed a one-credit course to provide training in a variety of fundamental research topics, including reproducibility, literature search, data management, data visualization, team science, and publishing. Here, NIH's proposed reproducibility training requirement for training grants guided the decision by the graduate program to make the fundamental research skills class mandatory for all doctoral students [10].

The class, which covered a wide variety of topics without a clear unifying theme, was not a satisfying instructional experience for us; likewise, attitudinal evaluations from students were mixed. This dissatisfaction with our existing course, combined with the release of the NIH rigor and reproducibility instruction guidelines in 2019, provided the impetus to overhaul our course curriculum to focus explicitly on rigor and reproducibility.

### Offerings

#### For-Credit Basic Science Class

The curriculum redesign resulted in a one-credit, required class for first-year biomedical PhD and MD/PhD students, called “Rigor and Reproducibility.” The current iteration of the class consists of eight 90-minute sessions. Following the NIH's Rigor and Reproducibility framework [13], we divided the class into four main components: rigor of the prior research, rigor of the proposed research, consideration of key biological variables, and authentication of key biological resources [13]. We based learning objectives on these guidelines and then employed a backward design to structure class topics around each learning objective [28]. Our course was designed around the following objectives; more information on specific classes is available in the syllabus [29]:

Students will be able to …

1. Outline and describe issues of replicability at each phase of the research process.
2. Appraise an article and identify strengths and weaknesses with regard to rigor and reproducibility.
3. Perform a comprehensive literature search and summarize the relevance of effective searching for rigorous research.
4. Outline the elements of best practices in research data management
5. Outline and explain the elements of computational reproducibility and explain their value.

All classes are taught by librarians with the exception of the class on authentication of key resources, which is taught by the director of our institutional scientific cores. The class is graded as pass/fail, with formative assessments primarily in the form of essays reflecting on how each class session's topic relates to the students' lived experiences as lab researchers. For example, when discussing randomization, we ask students to reflect on their experiences with randomization in their work and if it may have introduced problems with reproducibility. The format of our assessments allows us to get a sense of students' ability to critically engage with the material, especially as many of the topics discussed exist in a dialectic with pragmatic concerns of lab economics, efficiency, and the need for flexibility in exploratory research. A final exam asks students to respond to topics addressed in the class by identifying elements of computational and experimental reproducibility, building and running a literature search, and appraising an article for information on the rigor and reproducibility of the described study. To date all students have passed satisfactorily and their reflection assignments have demonstrated engagement with the topics and their own research practices.

#### Other Trainings

While the Rigor and Reproducibility course is the only full-semester, for-credit reproducibility course that we teach, we have offered other individual classes on rigor and reproducibility, both upon request and as part of a series of data classes that the library offers. Reproducibility classes were provided on request to dentistry postdocs, students in a clinical research intensive program, and to a general audience of participants through library educational programming. Computational reproducibility principles are also incorporated into required, for-credit courses in R programming for PhD and master's students. The library also offered a full-day reproducibility workshop and a ninety-minute introduction to reproducibility class. Whereas the full-day workshop was skills based (R programming and REDCap) and fairly well attended and well received, there were few attendees in the introductory class. This is reflective of our general findings that more people are interested in skills-based classes from the library [31].

### Assessment

A notable challenge we encountered was designing meaningful assessments for mastery of skills and topics discussed. The shift to short critical essays helped us to get a better sense of student engagement with topics compared to the previous multiple-choice tests; the essays also allow our learners to build on their experiences, reflecting and forming generalizable ideas around reproducibility [30].

In the most recent iteration for which there is an evaluation date, 2021, 12 out of 16 (75%) evaluation form responses said they would either recommend or highly recommend the class to others, and 4 of 16 (25%) said the class exceeded or far exceeded their expectations, with an additional 10 (63%) saying it met their expectations. These responses are overwhelmingly positive, and present a stark contrast to both the mixed reviews of the original iteration of this class and the reviews of other required courses.

### Lessons Learned

NYU's experience highlighted that partnering with relevant shareholders and departments in our medical center was pivotal for creating buy-in. Additionally, librarians' interest in providing teaching filled a need for our graduate biomedical and life sciences program that has allowed for further collaboration. We found that in the context of our for-credit class, a cohesive theme with time for reflective practice on the part of students was better received than stand-alone skills sessions. In the context of our broader medical center, skills-based trainings were better received than general introductory trainings on a broad topic, highlighting that education in reproducibility may need to be tailored around concrete skills to best serve our broader medical center community.

## SPENCER S. ECCLES HEALTH SCIENCES LIBRARY, UNIVERSITY OF UTAH

### Environment

The Spencer S. Eccles Health Sciences Library (EHSL), part of the University of Utah Libraries since 2018, is the library for the university's medicine, nursing, pharmacy, dentistry, and health professions schools and colleges, as well as the health care system (Table 1).

### Development

In 2015, due to a mutual interest in reproducibility and an interest in changing campus research culture, EHSL partnered with the vice president for research to host an interdisciplinary conference focused on how institutions can improve research reproducibility [24]. Following its success, we immediately launched a number of interdisciplinary efforts requested by our community to further the conversation and harness the momentum created by the conference. These efforts include a seminar series called Grand Rounds Research Reproducibility and a Reproducibility Short Course.

### Offerings

#### Grand Rounds Research Reproducibility

Grand Rounds Research Reproducibility (#UtahGRRR) [32] was a weekly seminar series designed to mimic traditional medical campus grand rounds series, but with an emphasis on interdisciplinarity and local experts. We envisioned #UtahGRRR to be a space where faculty, staff, students, and members of the public could share their work on reproducibility-themed topics and bring about new partnerships and cross-disciplinary collaborations. To ensure diverse representation among speakers, we purposefully solicited faculty, staff, and student speakers from a wide range of fields, including biostatistics, bioengineering, computer science, clinical medicine, philosophy, science journalism, and neuroscience, among others. We also offered continuing medical education (CME) credits to incentivize attendance. Topics in the grand rounds sessions ranged from the highly specific (error terms) to the more general (reproducibility in social science).

#### Short Course

The short course was developed in collaboration with the Department of Philosophy to provide academic credit, CME, or continuing education (CE) through the Medical Library Association (MLA). The general goal for the course was to prime people for our Research Reproducibility 2018 conference in order to enhance engagement for the conference. The course lasted four days with a fifth day being a 2018 conference on reproducibility [33].

We planned four major course outcomes:

1. Researchers and grantee attendees will increase awareness of research reproducibility issues.
2. Researchers and grantee attendees will acquire new knowledge, resources, or tools they can use to improve the reproducibility of their own research projects.
3. Librarians and information professionals will gain better knowledge and understanding of what trainees, researchers, and grantees need to know and want to know about research reproducibility.
4. Attendees will have concrete actions to take to improve reproducibility after the course.

The class was divided into morning and afternoon sessions, with mornings taught by a philosophy postdoc discussing both general and discipline-specific reasons that scientists fail at publishing reproducible results. The morning course outline:

- Day 1—Introduction
- Day 2—Case Studies
- Day 3—Problems Leading to Non-Reproducibility
- Day 4—Solutions to the Problem of Non-Reproducibility

The afternoon courses provided a hands-on learning opportunity in ways to implement reproducible practices discussed in the morning sessions. The afternoon sessions were taught by a librarian, Vicky Rampin (née Steeves). Each day of the course built on the material of the previous day, starting with Git, GitLab/GitHub, OpenRefine & R, Jupyter Notebooks, ReproZip & VisTrails, and ending with Open Science Framework to serve as a portfolio to connect the afternoon course lessons [34, 35].

### Assessment

The course had 34 individuals registered (5 external to the university, 5 students for academic credit, 20 internal to the university) either for CE credit or academic credit, and 25 attendees for some portion of the course. Attendee disciplines varied considerably, including machine learning, business, medicine, and digital humanities among others, and attracted participants from undergraduates to attending physicians. We received seven evaluations from the participants, a 12% response rate. Four rated the short course “excellent”; the remaining three respondents rated it “good.” All but one respondent said they learned more or much more than expected from the course. Feedback about the afternoon sessions was particularly positive, with several respondents noting that they would implement the skills they learned.

### Lessons Learned

#UtahGRRR worked well to establish a community on campus and share our campus experts' knowledge worldwide through session videos posted on YouTube. Though we did have a highly receptive environment for this work at Utah, the success we had was tied to people, not to the place. We consider our reproducibility efforts at the University of Utah a success, but a short-lived one. All of the EHSL faculty associated with the reproducibility efforts have subsequently left Utah, and initiatives did not endure. The volume and depth of programming and institutional leadership we were producing took massive, sustained effort by multiple faculty and staff at the library, and was made possible in part due to significant grant and other funding available. Ten or more staff and faculty were involved in these efforts to some degree, including film and digital content production, graphic design, and other behind-the-scenes efforts.

## HEALTH SCIENCE CENTER LIBRARIES, UNIVERSITY OF FLORIDA

### Environment

The Health Science Center Libraries (HSC Libraries) at the University of Florida (UF) support the faculty, staff, and students of UF Health and UF Health Jacksonville, including dentistry, medicine, nursing, pharmacy, public health and health professions, veterinary medicine, and the health care system (Table 1).

### Development

Beginning in 2018, new leadership at the HSC Libraries, coming from the University of Utah, introduced research reproducibility to outreach and educational efforts. Based on experiences of successful engagement with the research community at the University of Utah, we prioritized hiring of a new faculty librarian to support reproducibility and replicability in the health sciences and more broadly across the institution. Prior to hiring the new position, initial forays in education and training focused more narrowly on integrating education on the NIH Rigor and Reproducibility framework [13] into existing NIH training grants at UF and workshops on using reporting guidelines to translate research posters into research papers. Though efforts were initiated by the HSC Libraries, we quickly partnered with faculty from UF's Clinical & Translational Science Institute and other training grants and programs. After the new reproducibility librarian began in 2020, we significantly increased our efforts to build knowledge and skills around all aspects of reproducibility and replicability.

### Offerings

#### Guest Lectures

HSC librarians teach guest lectures on reproducibility in multiple settings, including a research conduct course, a summer research program, an institutional seminar series, and a credit-bearing course on literature searching and scientific communication. In offering these sessions, we partner with different groups on campus and tailor the content to the educational needs of each audience.

For example, in 2019, we added a rigor and reproducibility module to the required Responsible Conduct of Biomedical Research course for all students in the College of Medicine's graduate program in biomedical sciences. This session engages students in ethical decision-making around a reproducible research scenario, following the highly structured, team-based learning approach of the course [36]. For the Discovery Pathways Program, an optional summer program where medical students and potential MD/PhD students spend ten weeks working on research projects, our guest lecture focuses on reproducibility aspects related to research rigor and quality. The content includes an introduction to the reproducibility crisis and questionable research practices (p-hacking, HARKing, etc.), followed by an open discussion with students. Finally, in the one-credit course taught by HSC librarians for graduate students in biomedical sciences, “Finding Biomedical Research Information and Communicating Science,” one of the ten sessions focuses on how reproducibility affects the interpretation of published research. We discuss how different researchers may approach the same question and dataset in different ways, producing different results and interpretations [37].

#### Institutional Seminar Series

HSC librarians teach a session on reproducibility and replicability as part of a summer seminar series on Research Integrity and Responsible Conduct of Research (RCR), which was piloted in 2020, and open to all UF faculty, staff, and graduate students.

#### Library-Led Workshops

The reproducibility librarian teaches standalone workshops to bridge concepts in rigor and reproducibility with practical skills training, motivated by the perception that many researchers do not have capacity to engage with all of the literature on and apply best practices in statistics, questionable research practices, researcher degrees of freedom, etc. Thus, the workshop modules focus on entry ways into broadly useful practices or tools. For example, the “Data Organization in Spreadsheets” module [39] distills information from multiple sources [40–42] into practical guidance for organizing research files and tabular data in spreadsheets. These tips are immediately applicable by any researcher working with data, in contrast to instruction in data management plans and data deposition requirements that may not be of immediate use to student researchers.

Each module is structured as its own website with navigable slides and a set of curated links to additional training resources, reference guides, or deeper background literature [43]. Workshops are delivered synchronously over Zoom, and recordings are made available to registered attendees. The intent is to provide a persistent resource for researchers; thus, workshops begin by guiding learners on navigating to the lesson website. In keeping with open practices, all workshop materials are hosted on Github and archived to Zenodo [29], to maximize availability and reuse of the content as an open educational resource.

### Assessment

The reproducibility and replicability session in the RCR summer series was well received, with an average rating of 4.18 (on a 1-to-5 scale) from 56 ratings, in line with ratings for the entire series (average rating of 4.24 from 1,326 ratings). The overall series was highly popular as an alternative to self-guided web-based trainings for RCR accreditation, and feedback emphasized the importance of interactive exercises and small group discussions. The success of the pilot has spurred its continuation with a 2021 series [38].

### Lessons Learned

At University of Florida, having a dedicated librarian focused on reproducibility facilitated training and focus on reproducibility as a service area. In addition to having dedicated faculty to lead educational interventions, partnerships proved pivotal for University of Florida, particularly with the Clinical and Translational Science Institute and programs like the Discovery Pathways Program and the biomedical graduate program. Finally, University of Florida had success in filling skills gaps, as with library-led workshops on data organization and the Research Integrity and Responsible Conduct of Research class that fulfilled accreditation requirements, demonstrating that filling a concrete researcher need was a pathway to success for educational efforts.

## UNIVERSITY OF MINNESOTA

### Environment

The Health Sciences Library is part of the University of Minnesota (UMN) Libraries and serves the six colleges and schools associated with the health sciences: Medical School, School of Public Health, School of Dentistry, College of Pharmacy, College of Veterinary Medicine, and School of Nursing (Table 1).

### Development

The UMN Libraries have a well-established program of education and support for research data management, with an initial focus on NSF requirements in the hard sciences. Beginning in 2014, there was a broadening of these efforts to more directly address needs in the health sciences and the growing emphasis on data management and sharing from NIH and related funding agencies. Users articulated a need for pragmatic approaches to data management. Highly customized discussion-based sessions in labs and research groups were initiated in 2015 to help address this gap. These sessions focused on brief introductions to best practices of data management, followed by lab members mapping out their workflows as flowcharts and group discussions of the different workflows, which offered an opportunity for information sharing and peer-to-peer learning. This was a shift in focus of the libraries' approach to data management education, focusing on active data management practices in a workflow as opposed to centering the data management plan.

### Offerings

#### QuARRC: Quality Assurance Research Reproducibility Collaborative

As efforts in this area expanded, conversations grew beyond data management and sharing to more explicitly address issues of reproducibility and replicability. In 2016, two librarians were involved in QuARRC: Quality Assurance Research Reproducibility Collaborative, an NIGMS T32 Administrative Supplement Grant aimed to provide predoctoral training on quality assurance, data management, and good research practices. Partnering with faculty in the College of Veterinary Medicine and the Medical School, the project delivered a blend of interactive workshops, personalized consultations, panel presentations, and a two-day Data Carpentry workshop.

The relationship with QuARRC faculty continued after the grant ended. In 2019, we launched a new project to create online educational resources to help graduate, postdoctoral, and faculty researchers establish workflows and research practices that facilitate transparent, rigorous, and reproducible research. The course, developed with funding from the College of Veterinary Medicine's College of Online Learning Program, is designed as a mixture of videos, text, and interactive activities that covers topics from experimental design and statistics to quality assurance checkpoints to publication bias and situates these topics within a larger framework of open science.

#### Data and Software Carpentry Involvement

The Data Carpentry event organized through QuARRC marked the libraries' first involvement in organizing Carpentries events [44]. One librarian, Franklin Sayre, subsequently initiated a Software Carpentry pilot program in 2017 [45]. Through this pilot, two Software Carpentry workshops were hosted in 2017 and 2018 and included a focus on reproducible computation skills, including use of R, Bash, Git, and Python [45].

Since the pilot, the libraries' Carpentries Initiative led by Cody Hennessy has hosted 6 in-person 2-day workshops. The group has also experimented with different delivery modalities for Carpentries content, including workshop series ranging from 2 hours to 12 hours of instruction. This flexibility has been particularly valuable with the switch to remote learning in spring 2020. Since December 2018, over 150 hours of Carpentries instruction has reached over 400 learners at the University of Minnesota. A virtual Library Carpentry course was held through a series of weekly sessions in November and December 2020. This course, which was open to librarians throughout the United States, focused on developing computational and data management skills for information professionals and was attended by 42 librarians.

Outside of the Carpentries, workshops, seminars, and course-integrated instruction on reproducibility and data management have continued. These offerings now prominently feature discussions around reporting guidelines, preregistration, and questionable research practices, as well as core data management practices. While this evolution began organically, it allowed the libraries to connect data management skills and services to larger institutional goals of creating and promoting a culture of open scholarship on campus.

### Lessons Learned

As with other libraries in this paper, partnerships such as those with UMN's College of Veterinary Medicine and Medical School helped to achieve success in providing reproducibility training in QuARRC. Additionally partnering with the Carpentries allowed UMN to provide flexible training without the need for developing an in-house curriculum, a process that can be very time consuming and iterative. We were able to shorten and customize existing learning materials to meet the time needs and remote-learning needs of our community, highlighting the benefit of matching educational offerings to learner needs.

## VIRGINIA COMMONWEALTH UNIVERSITY

### Environment

Virginia Commonwealth University (VCU) is an urban, public, R1 university, located in Richmond, VA (Table 1). The VCU Libraries (VCUL) include a core branch library, a health sciences library, and a patient-facing health and wellness library. The research data librarian is a solo head of data services and functional liaison for data, working with subject liaisons in both libraries to serve both the medical and nonmedical campuses and their research data needs.

### Development

VCUL's RDM librarian is the lead and main librarian focusing on reproducibility and transparency. The RDM librarian works in collaboration with VCU's Data Science Lab and several divisions within the Office of the Vice President for Research's Division of Research Development. These collaborators requested that the RDM librarian respond to growing calls for education in reproducibility. Work on early requests led to further collaborations for RDM and reproducibility. VCUL's rigor and reproducibility partnerships center on training and teaching. In our events series before the pandemic we held an Open Science Day event to host activities for students to practice using repositories and writing FAIR-focused data management plans. Discussion groups have been an important out-of-class partnership as well. Specifically, the RDM librarian is a co-coordinator of our ReproducibiliTea journal club [46].

### Offerings

#### Guest lectures

One-shot and multipart guest lectures are a frequent approach to VCUL's reproducibility training. Guest lectures may be ad hoc or standing requests. In ad hoc requests at VCU, topics are typically an overview of the NIH policies, or introductory data management and documentation skills to promote transparency. The formats tend to be lecture heavy, interspersed with discussion prompts about the audience's experiences in their own projects, teams, publications, or grants. Guest lectures of this kind have been given in interdisciplinary research ethics, engineering, social work, and genomics courses. They have also been included as part of a non-credit research series in programs such as nursing, pharmacoeconomics, genomics, biomedical engineering, and health policy. In addition, a brief two- or three-slide mini-talk on the NIH's rigor and reproducibility policies is a frequent request to be included within the RDM librarian's broader talks to faculty or research staff. At the time of this article's writing, talks on the upcoming 2023 NIH data management and sharing policy will give a unique chance to talk about reproducibility as a strategy for getting “points” in grants, even though the data management and sharing plan is not in itself a scorable section.

VCU offers a broad, graduate-level Data Science 1 course for all disciplines (based in the Human Genetics department) that includes considerable rigor and reproducibility education. The RDM librarian has been a standing guest lecturer in the course, focusing on learning outcomes related to data workflow planning tasks, such as data documentation, file organization, permissions management, planning the text for tables and figures, and concepts around persistent identifiers, as well as lessons in using Open Science Framework or other generalist repositories.

#### Coteaching a credit course for NIH trainees

A summer Transparency and Reproducibility course is available for MD/PhD and PhD students to address the NIH's institutional trainee grant scoring on rigor and reproducibility. The RDM librarian teaches a module in this course and helps on other modules, and they are a credited co-instructor.

Course leaders formed an interprofessional team with faculty from multiple schools and units. A team of co-instructors organized our topics into five modules. Seven co-instructors came from medicine, biostatistics, research integrity, pharmacy, and computational genomics, in addition to the library. The RDM librarian serves as lead instructor on the module on publication-stage issues of data reporting and transparency, and has a secondary role in the data recording and analysis module.

The data reporting and transparency module had three learning objectives:

1. Explain common challenges to reproducible workflows, and how researchers are working toward increased reproducibility balanced with privacy.
2. Discuss elements needed during dissemination to promote reproducibility, and use those to consider what processes are needed to reach reproducibility goals.
3. Apply a transparent reporting rubric or checklist to articles to consider how existing studies could have been made more reproducible.

The course uses asynchronous and synchronous discussion activities for these three learning objectives. Readings and class discussions, critical appraisal of the Transparency and Openness in Publishing guidelines [47], and groupwork to apply one of the Equator Network checklists [48] about transparent reporting were employed to meet these objectives.

### Lessons Learned

At VCUL we found that discussion of topics with class groups was an effective way to foster communication and consider challenges in reproducibility across disciplines. Discussion prompts for reflection and sharing helped learners to think across labs and disciplines, since reproducibility is a broad concept without one-size-fits-all solutions, and implementation practices are project-specific. As with other libraries in this paper, we found that partnerships with other departments and subject librarians were essential to success in reproducibility education.

For us, and for others who might want to follow our approach, this means time to build relationships is important. Extra time has been needed to discuss with subject departments, co-instructors, and subject librarians to dig into where different groups have different needs. Thus, building in time to understand partners is important to growing programs beyond our initial generic reproducibility offerings.

## DISCUSSION

As demonstrated above, there are different ways to approach reproducibility education, but there are also commonalities. Key contextual issues at each institution that informed the specific instructional offerings include the impetus or motivation, the collaborators, and where appropriate, the funders and their goals. For ease of understanding, we summarize comparison of some key aspects of these contextual factors and the resulting offerings in Table 2.

**Table 2:** Summary of Rigor and Reproducibility Training at Five Academic Medical Libraries

	NYU Langone Health Sciences Library	Spencer. S. Eccles Health Sciences Library, University of Utah	Health Science Center Libraries, University of Florida	University of Minnesota	Virginia Commonwealth University
Impetus	NIH Requirements	Collaborator interest; NIH regulations	NIH Requirements; other demand for RCR and Reproducibility Training	Collaborator interest; NIH requirements	Collaborator interest; NIH reproducibility regulations
Collaborators	Graduate Biomedical Program, Institutional cores	Vice President for Research, College of Medicine and other departments, Department of Philosophy	College of Medicine and other liaison departments, Office of Research	Minnesota Supercomputing Institute, College of Medicine, College of Veterinary Medicine's Quality Central program	Office of Research; School of Medicine ADR's office; Data Science Lab core
Offerings	For-credit course; standalone workshops	Standalone events, guest lectures, for credit courses, conferences	Standalone workshops, guest lectures in for-credit courses	Standalone workshops; guest lectures in for-credit courses; workshop series	Standalone events; guest lectures in courses; for-credit coteaching
Funder	Partially Vilcek Institute of Graduate Biomedical Sciences	Office of Research Integrity - Department of Health and Human Services, Vice President for Research @ University of Utah, Center for Clinical and Translational Sciences @ U of U, MidContinental Region of the National Network of Libraries of Medicine, and Department of Philosophy @ U of U	Office of Research Integrity-Department of Health and Human Services, NIH T32 Supplemental Grant, NIH T32 Grant	NIH T32 Supplemental Grant; Internal funding sources	N/A

## GROWTH FROM COLLABORATIONS, STRENGTHENED BY MANDATES

Librarians engaging in reproducibility education started their work for a variety of reasons. In some cases, such as EHSL, UF's HSC, and VCU, engagement was primarily driven by a mixture of interest on the part of both individual librarians and their institutional collaborators. In the case of others, like University of Minnesota and NYU Langone Health Sciences Library, reproducibility teaching was strongly driven by national mandates. While institutions may have been driven by different stakeholder needs and motivations, the current situation is clear: reproducibility training is increasingly mandated by national funders. While it may be hard for any one department to tackle this problem, we have found that interdepartmental collaboration allows us to meet this challenge in a scalable, sustainable way.

In all cases discussed here, collaborating with other departments, from academic programs to administrative units, has helped make librarian-led teaching a success by providing a groundwork for curricular integration. We believe that the very nature of reproducibility as a cross-disciplinary issue makes a collaborative approach both necessary and effective. The wide range of skills and understanding needed to address different aspects of reproducibility education, from statistics to computation to authentication of biological resources, entails seeking expertise that is distributed across an institution. The library's institutional role in serving diverse departmental needs and stakeholders situates it well to work in coordinating and acting as a hub for service while bringing important skills around data management, establishing scientific premise, transparent reporting and publishing, and critical appraisal of literature.

### Wide range of course offerings, Broad Scope of Needs

The cases discussed here present a diverse scope of educational offerings, ranging from single-session guest lectures in classes to library-held multiday workshops to curriculum-integrated classes and CE work (Table 1). This highlights that there is no one-size-fits-all solution, but rather that interventions will need to reflect and respond to local needs. As noted above, many of our institutions' educational interventions grew out of collaborations and requests from institutional stakeholders. We found that developing local solutions to specific needs collaboratively led to meaningful relationships between librarians and relevant stakeholders, which in turn led to new opportunities for engagement.

Both in terms of patrons served and their own professional background, librarians need to be flexible in meeting the interdisciplinary challenge of reproducibility. Regarding students of educational interventions, as noted by UF, VCUL, and NYU Langone Health, learners may hail from various disciplines within the basic sciences as well as different backgrounds in the clinical world. Speaking across disciplines is a challenge, even among related fields within the basic sciences. Addressing this may mean working to incorporate a breadth of use cases, but also entails being transparent that not all topics will be equally relevant to all disciplines. In our experience, discussion about differences between fields and mitigating circumstances that impact adherence to reproducibility “best practices” can foster understanding among class participants and facilitators, as well as sharpen awareness of when different reproducibility practices will be most effective.

A related challenge is that in many cases the instructors themselves may not have lab experience. Transparency about the instructor's own background and openness to the participants' viewpoints helps build trust and foster insights. Given that reproducibility is more akin to a process or approach to research than a set of discrete skills, it is beneficial to generate an environment of trust to encourage learners to consider how different elements of a reproducible approach relates to their professional lives as researchers. As noted in the VCUL section above, concrete steps for implementing reproducible workflows will vary based on project-specific conditions. To address the need for specificity and to make training designs more flexible, a useful approach is to introduce concepts broadly and then discuss with students application to their own work. In both VCUL and NYU Langone Health's teaching, a discussion-oriented class was useful in helping researchers from different areas come to uncover and understand both opportunities and barriers to reproducible research as they applied in their area of work. Additionally, uncovering pragmatic limitations to reproducible workflows helped students to better engage and understand the complexity of reproducibility than may have been possible in a more didactic, “best-practice” oriented pedagogical intervention.

### Sustainability

Meeting the scope and breadth of need is time-intensive, which in turn creates challenges for the sustainability of new initiatives. As the EHSL case noted, projects that are heavily dependent on specific leaders can flounder should those individuals move to another institution or retire, or when their priorities shift. This issue is not limited to reproducibility projects and highlights a common difficulty in medical libraries. In the case of UF, the HSC Libraries were able to hire a full-time reproducibility librarian, which greatly facilitated allotting time to educational efforts in this area. Ultimately investment in reproducibility services will require library leadership buy-in and understanding of their local conditions as to the benefits and opportunity costs. Reproducibility support may align with strategic priorities such as research engagement, emerging data services, or improving scientific practices for the future. It is key to know whether these priorities are a valuable strategic fit for the library and institution.

One option to mitigate or avoid this issue is in working with stakeholders to broaden buy-in among departments and, when possible, curricula. Partnerships involving data librarians and subject specialist liaisons can be a way to expand topics and build cross-training to distribute instructional expertise and responsibilities so as to address sustainability. It may also be the case that as national funder mandates strengthen in this area, reproducibility education will be increasingly required and be more formalized by institutions, similar to computational cores or other centralized services.

### Limitations

The cases discussed in this paper reflect the experiences of academic medical libraries from different areas of the United States, in institutions ranging from private, nonprofit medical centers to flagship state universities, and as such any given library's experience cannot be indiscriminately generalized. Additionally, all cases presented here are recounted by the librarians who managed the reproducibility projects and facilitated interventions. As such, these descriptions are filtered through our personal perspectives of the experiences. Finally, all evaluation materials discussed were based on questionnaires generated internally to assess interventions rather than validated instruments. Accordingly, statements of student experiences are not comparable across contexts and may not be accurate measures of intervention effectiveness on future behavior.

### Future Directions/Global Recommendations

Several trends seem unlikely to abate in rigor and reproducibility education, providing both an opportunity and a challenge to librarians interested in working in this area:

1. There are growing requirements from funders, journals, and national institutions. The past several years have seen organizations like NIH and NSF require rigor and reproducibility training and data management plans, while journals have set requirements for data sharing. The new NIH Data Sharing and Management requirements, set to go into effect in 2023 [49], will create a further impetus for the inclusion of a substantial research data management component in rigor and reproducibility trainings, providing further opportunity for librarians to be involved.
2. This opportunity also brings a challenge for training and professional development among librarians. LIS graduate programs, and funders of training programs such as the National Networks of Libraries of Medicine (NNLM), can help address this need by incorporating “train-the-trainer” skills for librarians so that they can, in turn, help library users gain reproducibility and RDM skills. While some institutions have begun offering training to LIS students and working professionals, we expect educational demands and opportunities in reproducibility to continue to grow [50–52].
3. In keeping with broader research trends, librarians who teach rigor and reproducibility skills need to further incorporate and explicitly connect diversity, equity, and inclusion (DEI) principles into educational resources. Training will need to go beyond inclusion of sex as a biological variable to address issues of inequitable trial and study participation and their impact on generalizability, to promote the literature of ethical inclusion, and to teach about challenges around incentivization and privacy for open data initiatives [53, 54]. Librarians can also train users on the literature of biological variables and develop search hedges to improve future examination of under-addressed biological variables. Finally, there are many emerging challenges that libraries need to study relating to biases in machine learning, including racially biased training data, and other issues for DEI in research and data [55, 56].

## Data Availability

Data associated with this article are available at https://zenodo.org/communities/2021rigor_reproducibility_libraries_paper/.
