# Erratum to “Rigor and reproducibility instruction in academic medical libraries,” 2022;110(3):281-93.

**DOI:** 10.5195/jmla.2023.1853

**Published:** 2023-10-02

**Authors:** Katelyn Arnold

The PDF version of this article contained incorrect pagination from pages 281-290. The PDF has been updated to the correct pagination. The original article can be found via the DOI https://doi.org/10.5195/jmla.2022.1443.

